# Influence of Oxidative Stress Biomarkers and Genetic Polymorphisms on the Clinical Severity of Hydroxyurea-Free Senegalese Children with Sickle Cell Anemia

**DOI:** 10.3390/antiox9090863

**Published:** 2020-09-14

**Authors:** Fatou Gueye Tall, Cyril Martin, El hadji Malick Ndour, Camille Faes, Indou Déme Ly, Vincent Pialoux, Philippe Connes, Papa Madieye Gueye, Rokhaya Ndiaye Diallo, Céline Renoux, Ibrahima Diagne, Pape Amadou Diop, Aynina Cissé, Philomène Lopez Sall, Philippe Joly

**Affiliations:** 1 Laboratoire de Biochimie Pharmaceutique-FMPO, Universite Cheikh Anta Diop, Dakar BP 5005, Senegal; fatougueye.tall@ucad.edu.sn (F.G.T.); elhadjimalickndour@yahoo.fr (E.h.M.N.); gmadieye@yahoo.fr (P.M.G.); dabafr@yahoo.fr (R.N.D.); prpadiop@yahoo.fr (P.A.D.); aycisse@yahoo.fr (A.C.); plsall@yahoo.fr (P.L.S.); 2 Laboratoire Interuniversitaire de Biologie de la Motricité (LIBM) EA7424, Equipe Biologie Vasculaire et du Globule Rouge, Universite Claude Bernard Lyon 1, COMUE Lyon, 69100 Villeurbanne, France; camille.faes@univ-lyon1.fr (C.F.); vincent.pialoux@univ-lyon1.fr (V.P.); philippe.connes@univ-lyon1.fr (P.C.); celine.renoux@chu-lyon.fr (C.R.); 3 Centre Hospitalier National d’Enfants Albert Royer-Dakar, Dakar BP 5005, Senegal; cyril.martin@univ-lyon1.fr (C.M.); inddeme@yahoo.fr (I.D.L.); 4 Laboratoire d’Excellence sur le Globule Rouge (Labex GR-Ex), 75000 Paris, France; 5 Service Universitaire de Pédiatrie-FMPO, Universite Cheikh Anta Diop, Dakar BP 5005, Senegal; ibrahima.diagne@yahoo.fr; 6 UF Biochimie des Pathologies Erythrocytaires, Laboratoire de Biochimie et Biologie Moleculaire Grand-Est, Groupement Hospitalier Est, Hospices Civils de Lyon, 69500 Bron, France; 7 UFR des Sciences de la Santé–Universite Gaston Berger, Saint-Louis 32002, Senegal

**Keywords:** sickle cell anemia, hydroxyurea-free, alpha-thalassemia, HbF QTL, G6PD deficiency, clinical severity, oxidative stress parameters, oxidative stress polymorphisms

## Abstract

Oxidative stress would play a role in the pathophysiology of sickle cell anemia (SCA). We tested the impact of common SCA genetic modifiers (alpha-thalassemia, G6PD deficiency, HbF quantitative trait loci; QTL) and pro/antioxidant genes polymorphisms (*SOD2* rs4880, *XO* rs207454, *MPO* rs2333227) on oxidative stress biomarkers (AOPP, MDA, MPO, XO, MnSOD, CAT, GPx) and clinical severity in 301 Senegalese SCA hydroxyurea-free children at steady-state (median age 9.1 years, sex ratio H/F = 1.3). Plasma oxidative stress biomarkers were compared with those of a control group (AA). CAT activity, AOPP, and MDA levels were higher in SCA than in AA individuals while XO, GPX, and MnSOD activities were lower. The presence of alpha-thalassemia decreased MDA level and MPO activity but no effect of the HbF QTL or G6PD deficiency was observed. SCA children who experienced their first hospitalized complication before 3 years old had higher MnSOD and CAT activities than the other children while those with no hospitalized VOC in the previous 2 years presented higher GPX activity. Age of the first hospitalized complication and AOPP levels were affected by the *MPO* rs2333227 SNP. Our results suggest that alpha-thalassemia modulates oxidative stress in SCA, presumably because of a reduction in the MPO activity.

## 1. Introduction

Sickle cell anemia (SCA) is a genetic disorder characterized by a high inter-individual clinical variability partly related to the existence of various genetic modulators such as hemoglobin F (HbF) quantitative trait loci (QTL), alpha-thalassemia, and glucose-6-phosphate dehydrogenase (G6PD) deficiency [1]. Oxidative stress is enhanced in SCA and would also play a major role in the pathophysiology of the disease by promoting red blood cell (RBC) damage, inflammation, and endothelial-vascular dysfunction [1,2,3,4]. Elevated advanced oxidation protein products (AOPP) and malondialdehyde (MDA) concentrations have been consistently reported in patients with SCA, both at a systemic or RBC level [5,6,7,8,9,10,11,12]. As a consequence, it would be expected that RBC and plasma from patients with SCA exhibit decreased antioxidant capacities. However, rather inconsistent results were reported in the literature. For instance, Biswal et al. [11] reported lower superoxide dismutase (SOD), catalase (CAT) and glutathione peroxidase (GPX) activities in SCA children compared to controls. Mockesch et al. [7] found higher SOD activity in SCA than in healthy children and similar CAT and GPX activities in the two populations. Renoux et al. [12] described higher SOD and lower GPX activities but similar CAT activity in a group of SCA children compared to a control group. Finally, Faes et al. [13] found higher GPX activity in young adults with SCA compared to healthy individuals but no difference in SOD and CAT activities. These findings show that a clear picture regarding the activities of plasma antioxidant enzymes is difficult to depict in SCA.

One of the major concerns about these previous studies is that they were performed on relatively small groups of patients, some of whom being under hydroxyurea (HU) treatment that strongly modulates oxidative stress levels in SCA [14]. Moreover, the potential influence of classical SCA modifier genes on oxidative stress levels has not been taken into account, except by Renoux et al. [12] who reported decreased oxidative stress in SCA patients with alpha-thalassemia compared to those without. However, it remains unknown, for example, whether G6PD deficiency is associated or not with higher oxidative stress levels in SCA, as it is the case in the general population [15,16]. The progression of several chronic diseases has also been linked to antioxidant systems deficiencies with a potential modulation by specific single nucleotide polymorphisms (SNPs) [17]. However, no clear association between oxidative stress enzyme activities and clinical severity has been reported to date in SCA and the data about the role of these SNPs are very scarce in the literature.

In that context, the objectives of this work conducted in a large Senegalese cohort of HU-free children and adolescents with SCA were (i) to determine the effects of the three main SCA genetic modifiers (i.e., alpha-thalassemia, G6PD deficiency, and HbF QTLs) on plasma oxidative stress markers and pro/antioxidant enzymes activities, (ii) to test the associations between these markers and several indicators of clinical severity, and (iii) to study the effects of specific SNPs on genes encoding for some pro/antioxidant enzymes. Our results suggest that alpha-thalassemia modulates oxidative stress in SCA, presumably because of a reduction in the MPO activity.

## 2. Materials and Methods

### 2.1. Recruitment of the Cohort and Clinical Data Recording

The recruitment was carried out between January 2015 and December 2017 at the Albert Royer Children’s Hospital in Dakar. A total of 301 children and adolescents with homozygous sickle cell disease (SCA; SS genotype) were consecutively included during a routine follow-up visit (169 boys and 132 girls, median age 9.1 years). All patients were at steady state (no hospitalization or transfusion during the last 3 months) at the time of inclusion and none of them were treated with HU or attended a regular RBC exchange program.

The individual medical records were retrospectively reviewed to recover the age of the first hospitalized SCA complication, as well as the number of hospitalized vaso-occlusive crisis (VOC) and the occurrence of other complications (i.e., sepsis, osteonecrosis, osteomyelitis, stroke, acute splenic sequestration, and acute chest syndrome) in the 2 years preceding the inclusion. The study was approved by the Ethics Committee of the Cheikh Anta Diop University of Dakar (0079/2015/CER/UCAD) and all legal caregivers gave their consent, including for the genetic analyses. Twenty-five healthy children and adolescents were also recruited as controls for the determination of plasma levels of products and enzymes of oxidative stress.

### 2.2. Biochemical and Hematological Parameters

The following hematological and biochemical parameters were measured during the routine medical follow-up of the patients: (i) total hemoglobin (HB), white blood cells (WBC), platelets (PLT), and reticulocytes (RET) counts using a Sysmex XT-4000i device (System Corporation, Tokyo, Japan), (ii) lactate dehydrogenase (LDH), total and direct bilirubin (BIL), aspartate amino-transferase (ASAT) and C-reactive protein (CRP) using a BA-88 Mindray analyzer (Manwah, NJ, USA). The different hemoglobin fractions were quantified by cation exchange high-performance liquid chromatography (CE-HPLC) using a Variant II device and the beta-thalassemia short program (Biorad, Hercules, CA, USA).

### 2.3. Measurement of Plasma Oxidative Stress Markers

A blood sample collected in Ethylenediaminetetraacetic acid (EDTA) tube was immediately centrifuged at 3000× *g* rpm for 10 min at 4 °C and plasma aliquots were prepared and stored at −80 °C until analysis. Two global markers of oxidative stress were measured: AOPP with the semi-automated method developed by Witko-Sarsat et al. [18] and MDA using the spectrophotometric Ohkawa method [19] based on thiobarbituric acid reactions. The activities of two pro-oxidant enzymes (xanthine oxidase, XO; myeloperoxidase, MPO) and of three antioxidant enzymes (MnSOD, CAT, and GPX) were also measured. CAT activity was measured by the reaction of methanol and hydrogen peroxide (H_2_O_2_) which leads to formaldehyde formation using catalase as enzyme [20]. GPX activity represents the rate of NADPH elimination in NADP+ after addition of glutathione reductase, reduced glutathione and NADPH using H_2_O_2_ as substrate [21]. MnSOD activity was determined with the method of Beauchamp and Fridovich [22], modified by Oberley [23], and represents the degree of inhibition of the reaction between superoxide radicals, produced by a hypoxanthine-xanthine oxidase system, and nitroblue tetrazolium. XO activity was calculated by measuring spectrophotometrically the kinetic appearance of the complex formed by superoxide and 2-nitro-5-thiobenzoic acid (NTB) at 560 nm for 10 min [24]. Finally, MPO activity was measured by a semi-quantitative immunoassay using stabilized human anti-MPO antibodies (MPO, Human, clone 266-6K1, HM2164, Hycult Biotech). The MPO/anti-MPO complex was detected by spectrophotometry after addition of a 3,3′, 5,5′-tetramethylbenzidine solution (TMB, Sigma) with H_2_O_2_ as chromogenic substrate. Due to its immunological specificity, this method is considered as the gold-standard for measuring MPO activity in biological samples [25].

Samples were randomly allocated within each microplate. Laboratory personnel were blinded to the samples. All samples were assayed in duplicate. For any sample, if the within-individual coefficient of variation (CV) between duplicate samples was above 20%, we used the value that was closest to the mean concentration of the population tested. Values above four SDs from the mean of the population were defined as outliers and removed. Values below the limit of detection were removed from analyses for each assay. The intra-assay coefficients of variation (CV) and limits of detection of the assays are provided in Table 1.

### 2.4. Genotyping of SCA Modifiers and SNPs of Anti/Pro-Oxidant Enzymes Genes

The main alpha-thalassemia deletions (−3.7 Kb, −4.2 Kb, −20.5 Kb, MED and SEA) and *G6PD* deficient variants (Med, A- and Betica variants) were searched by a multiplex gap-PCR method [26] and dedicated in-house high resolution melting (HRM) protocols [27,28], respectively. Three HbF QTLs were genotyped: the so-called *XmnI* polymorphism in the promoter region of the ^G^gamma-globin gene (rs748214; *HBG2*:c.-211C > T) and two tag- SNPs in the *BCL11A* (rs1427407 G > T in intron 2) and *HMIP* (rs28384513 A > C) loci. As previously described [29], a composite HbF QTL score ranging from 0 to 6 and corresponding to the number of HbF-promoting alleles was calculated with these data. Finally, three SNPs previously described as affecting the activity of their respective oxidative stress enzyme were genotyped: rs4880 for the *SOD2* gene [30], rs207454 for the *XO* gene [31], and rs2333227 for the *MPO* gene [32,33,34].

Except for alpha-thalassemia deletions, all experiments were conducted with the Light Cycler 480^®^ device (Roche diagnostics, Meylan, France). The *XmnI* genotyping was done with a fluorescence resonance energy transfer (FRET) method while dedicated in-house HRM protocols were used for other SNPs.

### 2.5. Statistical Analyses

Continuous variables were reported as mean ± standard deviation (SD) and qualitative variables as number of patients (N). The four hemolytic parameters (i.e., RET, BIL, ASAT, and LDH) were resumed in one variable (hemolytic index–HI) using principal component analysis, as previously described [35,36]. Depending on the situation, unpaired Student’s *t*-tests, ANOVA, or Chi-square tests were employed. Statistical analyses were performed using SPSS (Statistical Package for Social Sciences) software version 22 (SPSS Inc., Chicago, IL, USA). The threshold of significance was defined at *p* < 0.05.

The levels of oxidative stress biomarkers were first compared between the SCA children and the healthy AA controls. Thereafter, oxidative stress parameters (as well as HI) were compared among SS children according to their clinical severity and to their HbF, G6PD, and alpha-thal status. Three indicators of clinical severity were used: age of the first hospitalized complication (<3 years old versus >3 years) and occurrence or not of VOC and acute chest syndrome during the last 2 years. Two classifications were tested for HbF: a first one using the HbF level measured by CE-HPLC (<5%, between 5% and 15%, and ≥15%) and a second one using the HbF-QTL score ((0–1), (2–3), and (4–6)).

A genetic dominant model was used to study the effects of the *SOD2*, *MPO,* and *XO* genetic polymorphisms on both oxidative stress (global markers and corresponding enzymes) and SCA clinical severity. Such a model distinguishes wild-type patients from those bearing the polymorphism at the heterozygous or homozygous state. The following indicators of clinical severity were tested: age of the first hospitalized complication (<3 years old versus >3 years), number of hospitalized VOC during the last 2 years, and occurrence or not of sepsis, osteonecrosis, osteomyelitis, stroke, acute splenic sequestration, and acute chest syndrome during the same period.

## 3. Results

### 3.1. Description of the SCA Population

The biological and clinical characteristics of our SCA cohort are presented in Table 2. The mean age at the time of inclusion was 9.7 years with a quite balanced sex ratio. About half of patients (146 out of 301) had their first hospitalized complication before the age of 3 years old. During the 2 years before inclusion, a majority of patients (176 out of 301) did not experience hospitalized VOC while the incidence of the other SCA complications was very low (from six for sepsis to 15 for osteomyelitis).

### 3.2. Comparison of Oxidative Stress Biomarkers between SCA and AA Patients

**Figure 1:**
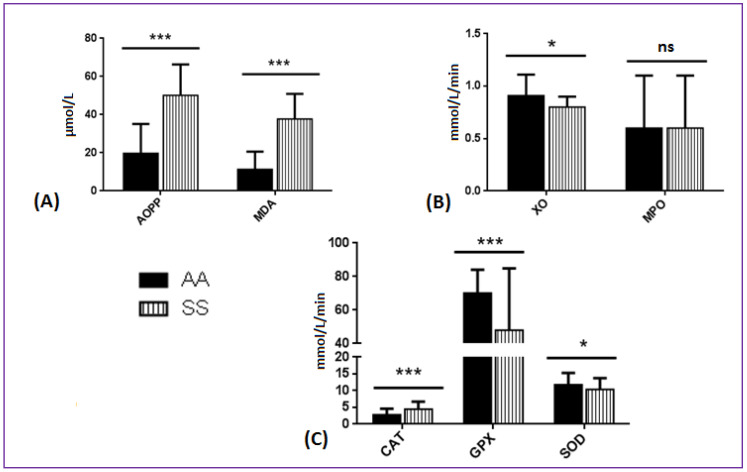
Comparison of oxidative stress products and enzymes between 301 SS children and 25 age-matched AA children; (**A**) oxidative stress products: AOPP (advanced oxidation protein products) and MDA (malondialdehyde); (**B**) pro-oxidant enzymes: XO (xanthine oxidase) and MPO (myeloperoxidase); (**C**) antioxidant enzymes: MnSOD (manganese super-oxide dismutase), CAT (catalase) and GPX (glutathione peroxidase). *: *p* < 0.05 at Student’s *t*-test; ***: *p* < 0.001 at Student’s *t*-test. ns: non significant.

Compared to AA patients, CAT activity and both AOPP and MDA levels were significantly elevated in patients with SCA. Conversely, XO, GPX, and MnSOD activities were significantly lower in SS compared to AA individuals (Figure 1, exact values in Supplemental data 1).

### 3.3. Effects of SCA Genetic Modifiers on Oxidative Stress Parameters and Hemolytic Index

**Figure 2:**
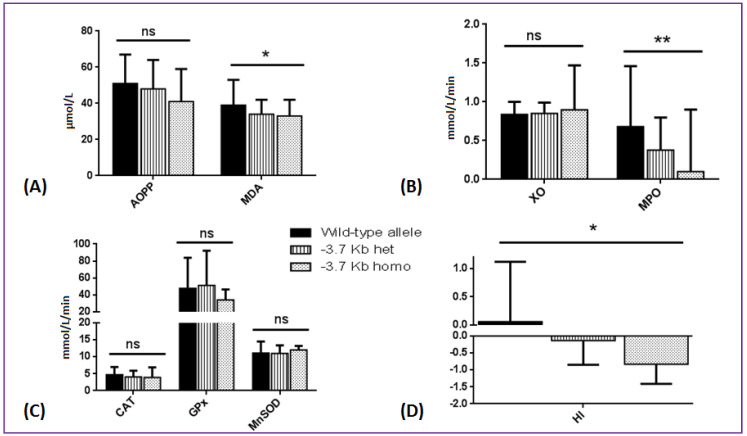
Comparison of oxidative stress biomarkers and hemolytic index according to the alpha-globin genotype in 301 SS children. (**A**) Oxidative stress products: AOPP (advanced oxidation protein products) and MDA (malondialdehyde); (**B**) pro-oxidant enzymes: XO (xanthine oxidase) and MPO (myeloperoxidase); (**C**) antioxidant enzymes: MnSOD (manganese superoxide dismutase), CAT (catalase), and GPX (glutathione peroxidase); (**D**) hemolytic index. *: *p* < 0.05; **: *p* < 0.01. ns: non significant.

Among the SCA patients, MDA concentration, MPO activity, and HI were significantly lower in presence of the −3.7 Kb deletion (i.e., the only alpha-thalassemia defect identified in our cohort), especially at the homozygous state. A similar trend (despite not reaching significance) was observed for AOPP (*p* = 0.18) while XO, CAT, GPX, and MnSOD activities were not affected by the alpha-globin genotype (Figure 2, exact values in Supplemental data 2). No effect of G6PD genotype, HbF level, or HbF-QTL score was observed on HI and oxidative stress parameters (Table 3).

### 3.4. Associations between Oxidative Stress Biomarkers and Indicators of Clinical Severity

Children who experienced their first hospitalized VOC before 3 years old had higher MnSOD (11.0 ± 3.2 vs. 9.7 ± 3.3 mmol/L/min; *p* = 0.001) and CAT (5.0 ± 2.5 vs. 4.1 ± 1.8 mmol/L/min; *p* = 0.002) activities than children who had their first complication at a later age. Higher GPX activity was observed for children with no hospitalized VOC during the last 2 years but no difference was observed for acute chest syndrome (Table 4). Regarding the other complications (Supplemental data 3), higher GPX activity and a tendency for greater AOPP levels (*p* = 0.04 and 0.06, respectively) were observed in the nine children with osteonecrosis compared to those without. Similarly, a tendency for lower MnSOD activity (*p* = 0.06) was observed for the eight children who developed acute splenic sequestration in the last 2 years.

### 3.5. Influence of SOD2, MPO, and XO Genetic Polymorphisms on SCA Complications and Oxidative Stress Parameters

These data are presented in Table 5. Age of the first hospitalized complication and AOPP levels were affected by the *MPO* rs2333227 SNP. The other oxidative stress SNPs studied had no effect on clinical severity. More surprisingly, the corresponding enzymatic activities (as well as AOPP and MDA levels) were not influenced by the presence or not of the genetic polymorphism.

## 4. Discussion

Our study confirms the higher plasma concentrations of global oxidative stress markers (i.e., MDA and AOPP) observed in children with SCA compared to healthy AA controls [6,11,12,37,38,39]. MDA and AOPP result from polyunsaturated fatty acid peroxidation and advanced protein oxidation, respectively, and their excessive concentration may reflect reactive oxygen species (ROS) overproduction [40]. Although Castilhos et al. [6] and Adelakun et al. [40] reported similar results, the lower or equivalent XO and MPO activities found in SS children compared to AA controls seem very surprising, considering the key role of these enzymes in hemolytic diseases [41]. In SCA, these two activities have been shown to increase under hypoxia-reoxygenation stresses [42,43]. The fact that SCA children included in this study were at steady-state (with more than half of them without any hospitalized VOC in the last 2 years) probably explain their non-elevated MPO and XO activities compared to AA patients. Although antioxidant enzymes are supposed to be overwhelmed by ROS in a SCA context, we observed higher CAT activity in the plasma of SS children compared to AA controls. These findings support those of Detterich et al. who demonstrated that oxidative stress can be (at least partly) compensated within the RBC or plasma of patients at steady-state [44]. Oxidative stress results from an imbalance between pro-oxidant and antioxidant activities. The rise of some antioxidant enzymes activities in SCA could be interpreted as an expected compensatory response to keep the oxidative stress system in a healthy equilibrium at steady-state. Since CAT has a better scavenging efficiency than GPX, its overproduction may be favored to face with the high H_2_O_2_ encountered in SCA [45,46]. To sum up, apart from AOPP and MDA levels which are constantly elevated in SCA, important differences in oxidative stress parameters can be observed from one study to another, depending on environmental, medical, and dietetical differences between cohorts. Obviously, HU-free patients in Africa can hardly be compared with children followed in France (for example) where HU is more and more prescribed early in life [47].

The primary effect of alpha-thalassemia is to limit hemolysis by lowering the intra-cellular HbS concentration. As a consequence, the decreased amount of free circulating hemoglobin and heme would reduce oxidative stress reactions and the production of ROS. It might also explain the decreased MPO activity in SCA children with alpha-thalassemia since free heme has been reported to be a strong stimulator of neutrophils activation [48]. Overall, alpha-thalassemia would act by lowering the oxidative burst input and not by stimulating antioxidant defenses. Conversely, neither the G6PD genotype nor the HbF status seems to modulate the oxidative stress parameters. Renoux et al. reported similar results but it could be argued that most of their G6PD deficient children were alpha-thalassemia carriers [12]. Such recruitment bias was not present in our study. Although debated [49], G6PD deficiency has been suggested to increase the risks for cerebral vasculopathy in SCA because it could favor enhanced oxidative stress and endothelial damages [50,51]. Our results do not support an association between G6PD deficiency and oxidative stress. Regarding the impact of HbF levels on oxidative stress, our results confirm those of Rusanova et al. [52] who did not observe any significant relationship between HbF levels and GPX and MnSOD enzymatic activities. Whether the lack of association between HbF levels and oxidative stress markers remains in adulthood, where the incidence of late complications (renal and cardiac disorders, pulmonary arterial hypertension, and retinopathy) is greater, is unknown.

In our cohort, CAT and MnSOD activities were higher in SS children who experienced their first hospitalized complication before 3 years old compared to children who developed their first complication at a later age. Considering that the occurrence of a first hospitalized VOC early in life is a marker of long-term severity, this finding would suggest that these two enzymes are chronically activated in the children with higher severity to modulate oxidative stress over a long period of time. Conversely, GPX activity would be more representative of the current severity of the disease since children with no hospitalized VOC had lower GPX activity than children with a positive history of VOC over the last 2 years. The lack of association between pro/antioxidant gene polymorphisms and the corresponding enzyme activities suggests a limited use of these SNPs in a SCA context. This would confirm the data of Crawford et al. who did not find any influence of several antioxidant enzymes SNPs in other diseases where oxidative stress plays a key role, such as diabetes, cardiovascular diseases and bladder cancer [17]. The *SOD2* Val-16-Ala polymorphism was studied many times in numerous diseases with very conflicting results. In SCA, it was recently associated with a lower MnSOD activity and a higher prevalence of acute splenic sequestration [30]. Quite interestingly, we also found a tendency for lower MnSOD activities for the eight children who recently developed an acute splenic sequestration but without any relation with the *SOD2* Val-16-Ala polymorphism. The *MPO* rs2333227 polymorphism was previously associated with more frequent sepsis in a cohort of SCA children [33], thus suggesting a deleterious effect. In our study, it was associated with higher AOPP levels (enhanced oxidative stress) but also with a delayed age of first complication which would rather suggest a protective effect. These contradictory results do not allow drawing any conclusion about the role of this SNP in SCA.

Many studies previously investigated oxidative stress markers in SCD populations partially HU-treated [7,12,44,53]. However, their conclusions could be challenged since HU treatment has been shown to modulate oxidative stress [14]. On the other hand, the few studies performed in HU-free populations focused on a limited number of patients and/or a limited number of oxidative stress biomarkers [9,13,54,55,56,57,58,59]. It was therefore difficult to draw definitive conclusions about the impact of oxidative stress in SCD pathophysiology, independently of the effects of various treatments. The present study is the largest one (301 children included) conducted in a HU-free SCD population. It has thus some advantages to test the effects of oxidative stress in SCD. Moreover, it is the only one addressing the relationships between oxidative stress, clinical severity, and so many genetic polymorphisms since we tested both the three common SCD genetic modifiers (alpha-thal, G6PD deficiency, and HbF) and common oxidative stress polymorphisms in different genes. Nevertheless, because of the restricted resources in Senegal compared to European and US countries, some clinical exams are difficult to make in clinical routine (transcranial doppler velocities measurements or echocardiography for the measurements of cerebral vasculopathy and pulmonary hypertension, respectively). Consequently, some possible associations between oxidative stress markers and specific acute/chronic complications could not be addressed and it is probably the main disadvantage of our study.

## 5. Conclusions

Our work confirms the modulatory effects of alpha-thalassemia on oxidative stress in SCA, presumably by a reduction in the MPO activity. Conversely, neither the other classical genetic modifiers (G6PD, HbF) nor the oxidative stress SNPs seem to have a significant impact, at least during childhood. These observations will have to be confirmed and completed in another pediatric cohort but also mostly in adults since it is highly conceivable that the impact of oxidative stress in SCA would increase with age.

## Figures and Tables

**Table 1:** Intra-assay coefficients of variation (CV) and limits of detection of the oxidative stress assays.

Assays	Intra-Assay CV	Limit of Detection
AOPP	5.20%	2 µmol/L
MDA	4.23%	1 µmol/L
GPX	6.30%	2 mmol/L/min
Catalase	5.49%	0.1 mmol/L/min
SOD	6.35%	0.2 mmol/L/min
MPO	8.12%	0.02 mmol/L/min
XO	3.41%	0.01 mmol/L/min

**Table 2:** Overall clinical and biological characteristics of the pediatric sickle cell anemia (SCA) cohort.

Clinical and Biological Parameters	Mean (±SD) or N	Range (min–max)
**Epidemiological parameters**		
Inclusion age (years)	9.7 ± 4.6	2.0–22.9
Sex ratio (M/F)	170/131	/
**Age of first SCA complication**		
<3 years	146	/
>3 years	155	/
**Hospitalized VOC in last 2 years**		
None	176	/
At least one	125	/
**Other complications in last 2 years (No/Yes)**		
Osteomyelitis	286/15	/
Osteonecrosis	292/9	/
Stroke	289/12	/
Acute splenic sequestration	293/8	/
Sepsis	295/6	/
Acute chest syndrome	290/11	/
**Biological parameters**		
WBC count (10^3^/L)	14.3 ± 4.3	4.2–28.5
Hb (g/dL)	7.8 ± 1.1	5.5–12.0
Reticulocytes count (10^3^/L)	330 ± 166	28–1117
Reticulocytes (%)	12.0 ± 5.6	0.6–36.5
Platelets count (10^3^/L)	449 ± 136	135–945
HbF (%)	9.5 ± 5.1	1.1–26.8
ASAT (UI/L)	61 ± 34	20–341
Total bilirubin (mg/dL)	44 ± 24	7–107
Direct bilirubin (mg/dL)	23 ± 15	2–68
LDH (UI/ L)	940 ± 499	148–3318
CRP (mg/L)	4.6 ± 5.4	0.1–34.6

SCA: sickle cell anemia; VOC: vaso-occlusive crisis; WBC: white blood cell; Hb: hemoglobin; ASAT: aspartate amino-transferase; LDH: lactate dehydrogenase; CRP: C-reactive protein; AOPP: advanced oxidation protein products; MDA: malondialdehyde; SOD: superoxide dismutase; MPO: myeloperoxidase; XO: xanthine oxidase; CAT: catalase; GPX: glutathione peroxidase.

**Table 3:** Biomarkers of oxidative stress and hemolysis index for the 301 patients with SCA according to the HbF and G6PD status.

	HbF QTLs (0–6)			*p* *	HbF Level (%)			*p*	G6PD Genotype			*p*
	(0–1) n = 51	(2–3) n = 195	(4–6) n = 55		<5 n = 68	5–15 n = 185	≥15 n = 48		Wild n = 249	Het n = 25	Mute n = 27	
**Oxidative stress products**												
AOPP (µmol/L)	48 ± 16	51 ± 17	49 ± 14	0.58	51 ± 17	50 ± 16	48 ± 15	0.68	50 ± 16	49 ± 17	50 ± 16	0.89
MDA (µmol/L)	37 ± 8	38 ± 10	38 ± 23	0.82	37 ± 11	38 ± 14	39 ± 13	0.63	38 ± 14	37 ± 7	37 ± 7	0.90
**Pro-oxidant enzymes**												
XO (mmol/L/min)	0.84 ± 0.16	0.86 ± 0.16	0.84 ± 0.16	0.87	0.85 ± 0.18	0.84 ± 0.15	0.87 ± 0.17	0.56	0.84 ± 0.16	0.86 ± 0.16	0.84 ± 0.10	0.83
MPO (mmol/L/min)	0.6 ± 0.7	0.6 ± 0.9	0.5 ± 0.5	0.76	0.62 ± 0.86	0.64 ± 0.73	0.50 ± 0.50	0.47	0.6 ± 0.7	0.5 ± 0.5	0.6 ± 0.5	0.50
**Antioxidant enzymes**												
Catalase (mmol/L/min)	4.2 ± 1.2	4.7 ± 2.3	4.3 ± 2.0	0.22	4.7 ± 1.8	4.6 ± 2.4	4.2 ± 1.7	0.51	4.6 ± 2.2	3.8 ± 1.7	4.6 ± 1.8	0.24
GPX (mmol/L/min)	46.6 ± 34.4	49.3 ± 38.0	45.5 ± 35.5	0.75	49.4 ± 41.4	47.5 ± 36.0	48.8 ± 33.2	0.93	48.3 ± 37.0	53.9 ± 38.3	40.8 ± 32.0	0.42
SOD (mmol/L/min)	11.1 ± 2.6	11.1 ± 3.3	11.3 ± 3.4	0.73	10.9 ± 2.7	11.5 ± 3.4	10.4 ± 2.8	0.07	10.3 ± 3.26	10.8 ± 3.1	11.5 ± 3.4	0.31
**Hemolytic index**	0.07 ± 1.06	−0.02 ± 0.97	0.01 ± 1.05	0.84	0.03 ± 0.86	0.05 ± 1.09	−0.24 ± 0.81	0.19	−0.01 ± 1.02	0.10 ± 0.90	0.02 ± 0.92	0.87

AOPP: advanced oxidation protein products; MDA: malondialdehyde; XO: xanthine oxidase; MPO: myeloperoxidase; GPX glutathione peroxidase; SOD: superoxide dismutase; QTL: quantitative trait loci; n: number of patients. Mean values ± standard deviation. *: ANOVA test.

**Table 4:** Hemolytic index and biomarkers of oxidative stress for the 301 patients with SCA according to their clinical complications.

	Age of First Complication			Hospitalized VOC in Last 2 Years			Acute Chest Syndrome in Last 2 Years		
	<3 years n = 146	>3 years n = 155	*p*	None n = 176	At Least 1 n = 125	*p*	None n = 290	At Least 1 n = 11	*p*
**Oxidative stress products**									
AOPP (µmol/L)	49 ± 16	51 ± 16	*0.15*	50 ± 16	51 ± 16	*0.48*	50 ± 16	54 ± 20	*0.46*
MDA (µmol/L)	38 ± 16	37 ± 10	*0.53*	38 ± 14	37 ± 11	*0.51*	38 ± 13	42 ± 10	*0.28*
**Pro-oxidant enzymes**									
XO (mmol/L/min)	0.84 ± 0.15	0.85 ± 0.16	*0.88*	0.84 ± 0.16	0.84 ± 0.16	*0.85*	0.85 ± 0.16	0.85 ± 0.18	*0.89*
MPO (mmol/L/min)	0.6 ± 0.7	0.6 ± 0.7	*0.98*	0.6 ± 0.7	0.6 ± 0.7	*0.75*	0.62 ± 0.74	0.50 ± 0.51	*0.60*
**Antioxidant enzymes**									
MnSOD (mmol/L/min)	11.0 ± 3.2	9.7 ± 3.3	*0.001*	10.6 ± 3.3	10.0 ± 3.2	*0.19*	11.1 ± 3.2	12.5 ± 1.8	*0.14*
CAT (mmol/L/min)	5.0 ± 2.5	4.1 ± 1.8	*0.002*	4.6 ± 2.4	4.4 ± 1.7	*0.46*	4.5 ± 2.2	5.2 ± 2.4	*0.36*
GPX (mmol/L/min)	49.0 ± 38.9	47.3 ± 34.7	*0.67*	52.4 ± 35.2	42.0 ± 38.1	*0.01*	48.1 ± 37.0	47.7 ± 32.5	*0.97*

AOPP: advanced oxidation protein products; MDA: malondialdehyde; XO: xanthine oxidase; MPO: myeloperoxidase; MnSOD: manganese superoxide dismutase; CAT: catalase; GPX: glutathione peroxidase; n: number of patients. Mean values ± standard deviation.

**Table 5:** Influence of *SOD2*, *MPO*, and *XO* genetic polymorphisms on SCA complications and oxidative stress parameters.

	*SOD2* (rs4880)			*MPO* (rs2333227)			*XO* (rs207454)		
	Wild n = 132	Het + Mute n = 169	*p*	Wild n = 163	Het + Mute n = 138	*p*	Wild n = 142	Het + Mute n = 159	*p*
Quantitative clinical parameters *									
Age first complication (years)	4.4 ± 3.4	3.8 ± 3.2	*0.12*	3.7 ± 3.0	4.5 ± 3.5	*0.04*	4.0 ± 3.2	4.0 ± 3.2	*0.86*
Number of hospitalized VOC (last 2 years)	0.80 ± 1.3	0.69 ± 1.1	*0.43*	0.77 ± 1.3	0.71 ± 1.1	*0.69*	0.85 ± 1.4	0.65 ± 1.1	*0.16*
Other clinical parameters (No/Yes) **									
Osteomyelitis	124/8	162/7	*0.75*	153/10	133/5	*0.31*	133/9	153/6	*0.30*
Osteonecrosis	129/3	163/6	*0.51*	157/6	135/3	*0.44*	137/5	158/1	*0.07*
Stroke	124/8	165/4	*0.10*	156/7	133/5	*0.76*	137/5	152/7	*0.42*
Acute splenic sequestration	128/4	165/4	*0.72*	157/6	136/2	*0.23*	137/5	156/3	*0.37*
Sepsis	129/3	166/3	*0.75*	160/3	135/3	*0.83*	137/5	158/1	*0.07*
Acute chest syndrome	126/6	164/5	*0.53*	156/7	134/4	*0.52*	134/8	156/3	*0.08*
**Oxidative stress parameters**									
AOPP (µmol/L)	51.2 ± 16.4	49.4 ± 16.1	*0.33*	48.1 ± 15.5	52.6 ± 16.8	*0.02*	49.9 ± 15.1	50.3 ± 17.2	*0.83*
MDA (µmol/L)	37.6 ± 10.7	37.9 ± 14.7	*0.87*	38.1 ± 14.9	37.2 ± 10.5	*0.54*	37.9 ± 9.8	37.6 ± 15.4	*0.85*
XO (mmol/L/min)	Not calculated			Not calculated			0.57 ± 0.65	0.67 ± 0.79	*0.38*
MPO (mmol/L/min)	Not calculated			0.84 ± 0.15	0.84 ± 0.16	*0.93*	Not calculated		
MnSOD (mmol/L/min)	10.9 ± 2.9	11.1 ± 3.4	*0.22*	Not calculated			Not calculated		

*: Student’s *t*-test; **: Chi-square test. AOPP: advanced oxidation protein products; MDA: malondialdehyde; XO: xanthine oxidase; MPO: myeloperoxidase; MnSOD: manganese superoxide dismutase; n: number of patients. Mean values ± standard deviation.

## Supplementary Materials

The following are available online at https://www.mdpi.com/2076-3921/9/9/863/s1, Table S1: Biomarkers of oxidative stress in SS children and AA controls, Table S2: Biomarkers of oxidative stress and hemolytic index for the 301 patients with SCA according to the alpha-thalassemia genotype and Table S3: Biomarkers of oxidative stress for the 301 patients with SCA according to the main clinical complications on the last 2 years.
