# Supplementary material for: Influence of Oxidative Stress Biomarkers and Genetic Polymorphisms on the Clinical Severity of Hydroxyurea-Free Senegalese Children with Sickle Cell Anemia
Source: Antioxidants (Basel). 2020 Sep 14;9(9):863. doi: 10.3390/antiox9090863 (PMC7555380; doi:10.3390/antiox9090863)
Supplement: Supplementary file 1 [file antioxidants-09-00863-s001.zip › Supplemental data 1_v.5.pdf]

**Supplemental data 1. Biomarkers of oxidative stress in SS children and AA controls.**

| Markers | SS (N = 301) | AA (N = 25) | *p |
|---|---|---|---|
| **AOPP (µmol/L)** | 50.1 ± 16.2 | 19.8 ± 15.3 | *< 0.001* |
| **MDA (µmol/L)** | 37.8 ± 13.1 | 11.3 ± 9.3 | *< 0.001* |
| **XO, mmol/L/min** | 0.80 ± 0.1 | 0.91 ± 0.2 | *0.01* |
| **MPO, mmol/L/min** | 0.6 ± 0.5 | 0.6 ± 0.5 | *0.7* |
| **CAT, mmol/L/min** | 4.5 ± 2.2 | 2.8 ± 1.8 | *< 0.001* |
| **GPX, mmol/L/min** | 48.1 ± 36.8 | 70.1 ± 14.0 | *< 0.001* |
| **SOD, mmol/L/min** | 10.4 ± 3.3 | 11.8 ± 3.5 | *0.04* |

AOPP: advanced oxidation protein products; MDA: malonedialdehyde; XO: xanthine oxidase; MPO: myéloperoxidase; CAT: catalase; GPX: glutathion peroxidase; SOD: superoxide dismutase; N: number of patients. Mean values ± standard deviation.
