# Supplementary material for: Influence of Oxidative Stress Biomarkers and Genetic Polymorphisms on the Clinical Severity of Hydroxyurea-Free Senegalese Children with Sickle Cell Anemia
Source: Antioxidants (Basel). 2020 Sep 14;9(9):863. doi: 10.3390/antiox9090863 (PMC7555380; doi:10.3390/antiox9090863)
Supplement: Supplementary file 1 [file antioxidants-09-00863-s001.zip › Supplemental data 2_v.5.pdf]

**Supplemental data 2: Biomarkers of oxidative stress and hemolytic index for the 301 patients with SCA according to the alpha-thalassemia genotype.**

| | Alpha-thalassemia | | | p |
|---|---|---|---|---|
| | Wild<br>n = 235 | Het<br>n = 59 | Mute<br>n = 7 | |
| **Oxidative stress products** | | | | |
| AOPP (µmol/L) | 51 ± 16 | 48 ± 16 | 41 ± 18 | *0.18* |
| MDA (µmol/L) | 39 ± 14 | 34 ± 8 | 33 ± 9 | *0.03* |
| **Pro-oxidant enzymes** | | | | |
| XO (mmol/L/min) | 0.84 ± 0.16 | 0.85 ± 0.14 | 0.90 ± 0.57 | *0.58* |
| MPO (mmol/L/min) | 0.68 ± 0.78 | 0.38 ± 0.42 | 0.10 ± 0.8 | *0.003* |
| **Anti-oxidants enzymes** | | | | |
| Catalase (mmol/L/min) | 4.7 ± 2.3 | 4.1 ± 1.8 | 3.8 ± 3.0 | *0.13* |
| GPX (mmol/L/min) | 47.8 ± 36.1 | 51.2 ± 40.9 | 34.0 ± 12.5 | *0.48* |
| SOD (mmol/L/min) | 11.1 ± 3.4 | 11.0 ± 2.4 | 12.0 ± 1.2 | *0.11* |
| **Hemolysis index** | 0.06 ± 1.06 | - 0.13 ± 0.72 | - 0.83 ± 0.58 | *0.035* |

AOPP: advanced oxidation protein products; MDA: malonedialdehyde; XO: xanthine oxidase; MPO: myeloperoxidase; GPX: glutathion peroxidase; SOD: superoxide dismutase; n: number of patients. Mean values +/- standard deviation.
