# Supplementary material for: Influence of Oxidative Stress Biomarkers and Genetic Polymorphisms on the Clinical Severity of Hydroxyurea-Free Senegalese Children with Sickle Cell Anemia
Source: Antioxidants (Basel). 2020 Sep 14;9(9):863. doi: 10.3390/antiox9090863 (PMC7555380; doi:10.3390/antiox9090863)
Supplement: Supplementary file 1 [file antioxidants-09-00863-s001.zip › Supplemental data 3_v.5.pdf]

Supplemental data 3: Biomarkers of oxidative stress for the 301 patients with SCA according to the main clinical complications on the last 2 years

	Osteonecrosis			Sepsis			Stroke			Acute splenic sequestration			Osteomyelitis		
	No n = 292	Yes n = 9	*p*	No n = 295	Yes n = 6	*p*	No n =289	Yes n =12	*p*	No n = 293	Yes n = 8	*p*	No n = 286	Yes n = 15	*p*
**Oxidative stress products**															
AOPP (µmol/L)	50 ± 16	60 ± 19	*0.06*	50 ± 16	45 ± 17	*0.52*	50 ± 16	51 ± 24	*0.93*	50 ± 16	49 ± 5	*0.63*	50 ± 16	47 ± 10	*0.34*
MDA (µmol/L)	38 ± 13	35 ± 11	*0.78*	38 ± 13	31 ± 9	*0.17*	38 ± 13	35 ± 11	*0.48*	38 ± 13	40 ± 7	*0.57*	38 ± 13	33 ± 10	*0.16*
**Pro-oxidant enzymes**															
XO (mmol/L/min)	0.84 ± 0.16	0.86 ± 0.10	*0.80*	0.84 ± 0.16	0.90 ± 0.12	*0.43*	0.84 ± 0.16	0.84 ± 0.16	*0.90*	0.84 ± 0.16	0.94 ± 0.11	*0.11*	0.85 ± 0.17	0.85 ± 0.14	*0.98*
MPO (mmol/L/min)	0.6 ± 0.7	0.6 ± 0.6	*0.93*	0.6 ± 0.7	0.3 ± 0.2	*0.29*	0.6 ± 0.7	0.4 ± 0.3	*0.22*	0.6 ± 0.7	0.4 ± 0.4	*0.40*	0.6 ± 0.7	0.8 ± 0.9	*0.37*
**Anti-oxidant enzymes**															
MnSOD (mmol/L/min)	10.3 ± 3.3	10.8 ± 2.5	*0.64*	10.4 ± 3.3	11.4 ± 2.5	*0.47*	10.4 ± 3.4	10.1 ± 2.7	*0.74*	10.4 ± 3.3	8.2 ± 3.0	0.06	10.4 ± 3.4	10.1 ± 3.7	*0.72*
Catalase (mmol/L/min)	4.5 ± 2.2	5.0 ± 2.2	*0.58*	4.5 ± 2.2	4.0 ± 1.4	*0.49*	4.6 ± 2.2	4.2 ± 1.9	*0.61*	4.6 ± 2.2	4.4 ± 2.2	*0.86*	4.6 ± 2.2	4.3 ± 1.7	*0.50*
GPX (mmol/L/min)	47.3 ± 36	72.6 ± 53.8	*0.04*	48.3 ± 39.0	40.1 ± 25.0	*0.59*	48.2 ± 37.1	45.8 ± 29.4	*0.82*	48.6 ± 37.1	31.3 ± 14.0	*0.19*	48.0 ± 35.4	50.4 ± 59.2	*0.81*

AOPP: advanced oxidation protein products; MDA: malonedialdehyde; XO: xanthine oxidase; MPO: myeloperoxidase; MnSOD: manganese superoxide dismutase; GPX: glutathion peroxidase; n: number of patients. Mean values ± standard deviation.
